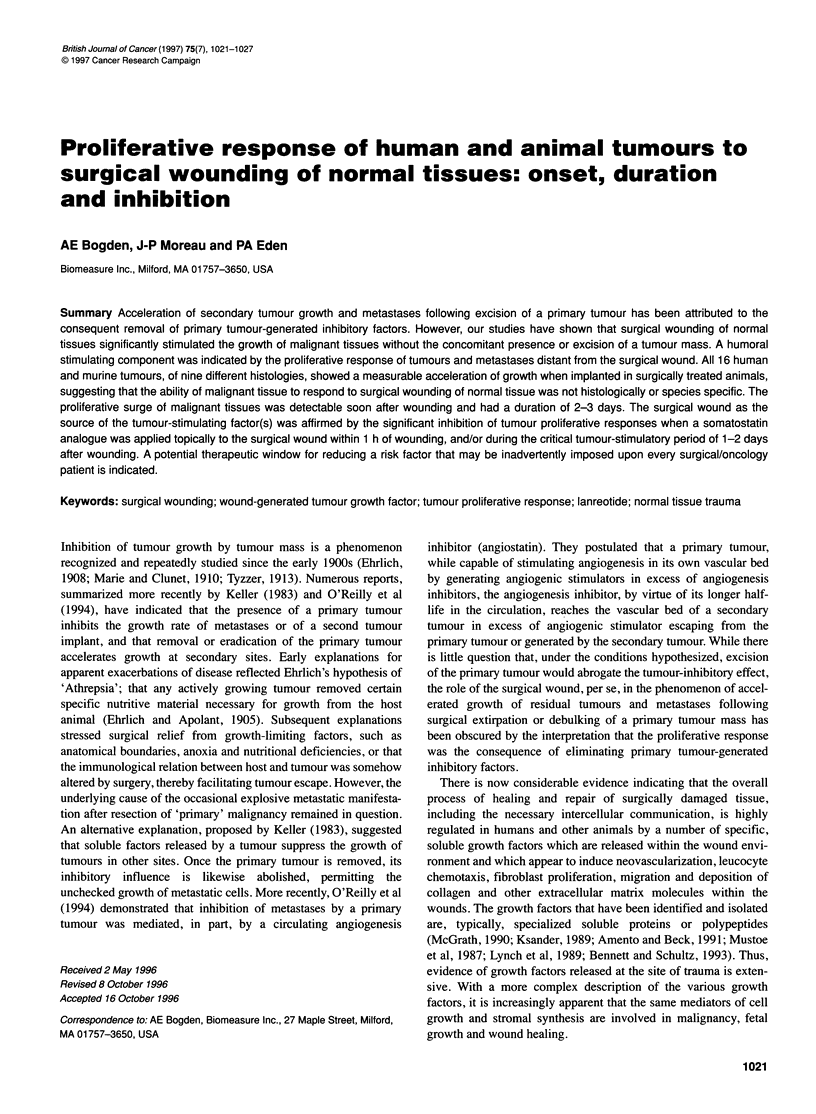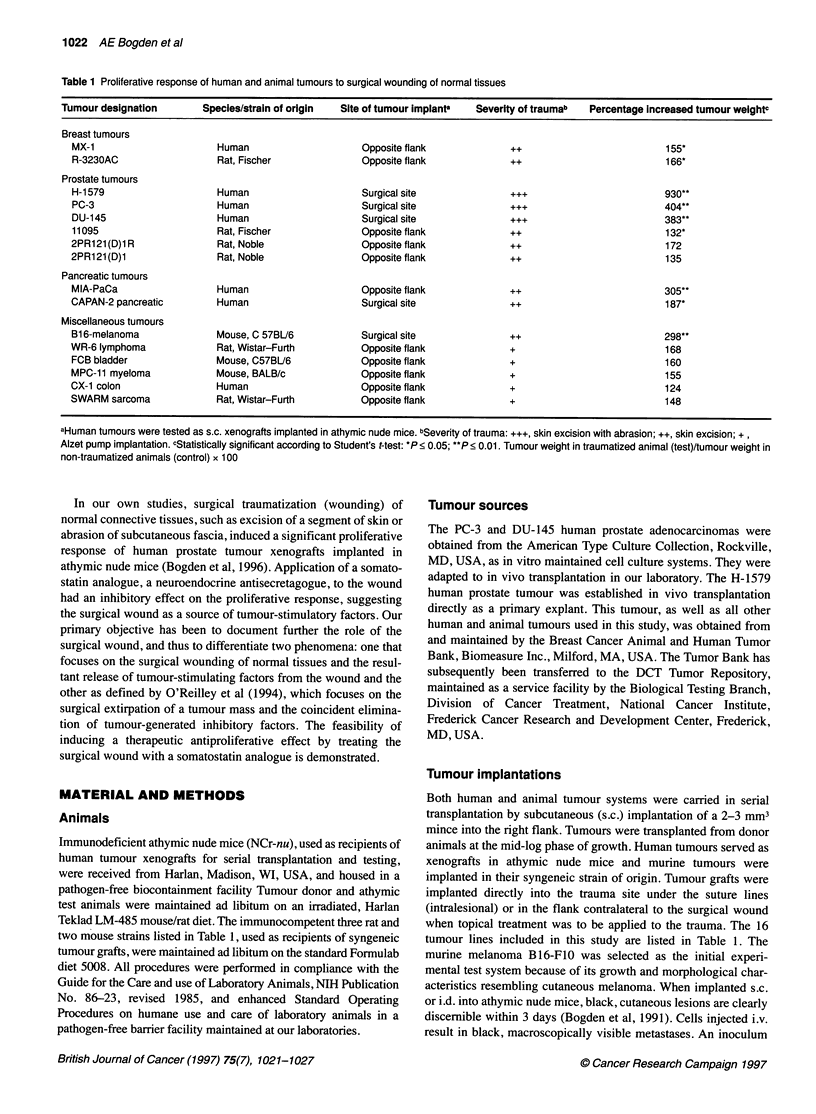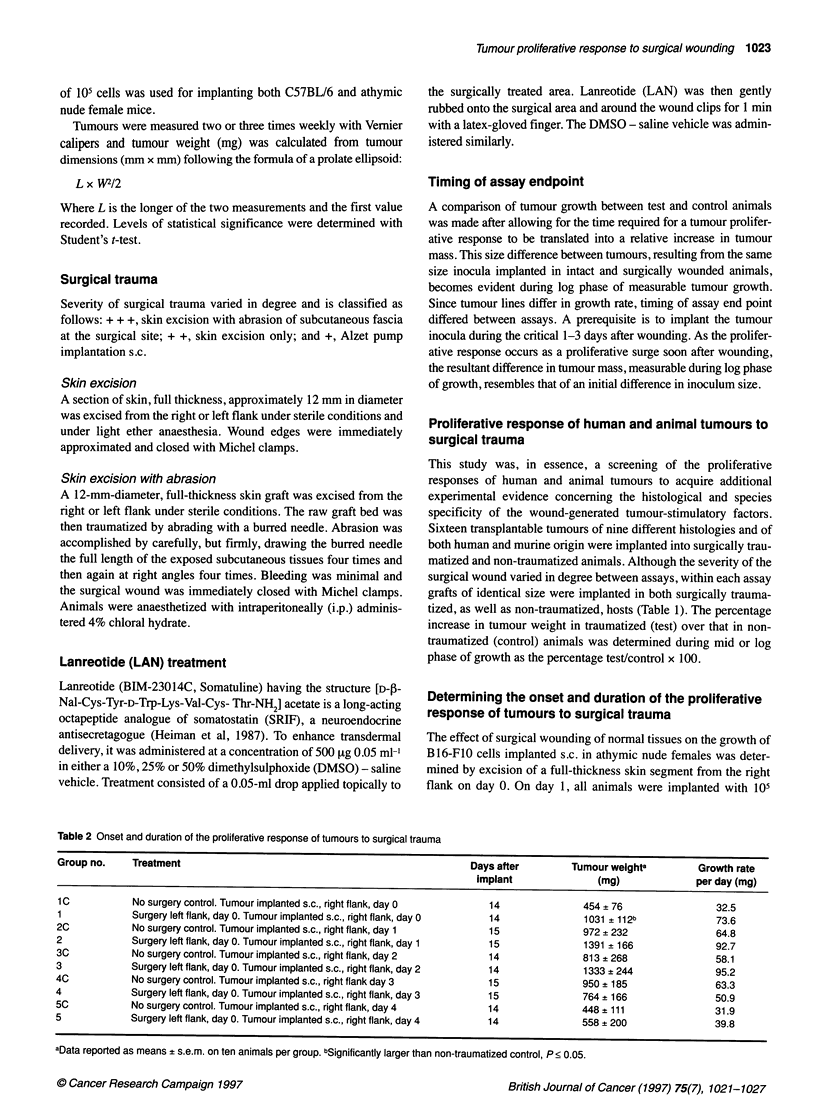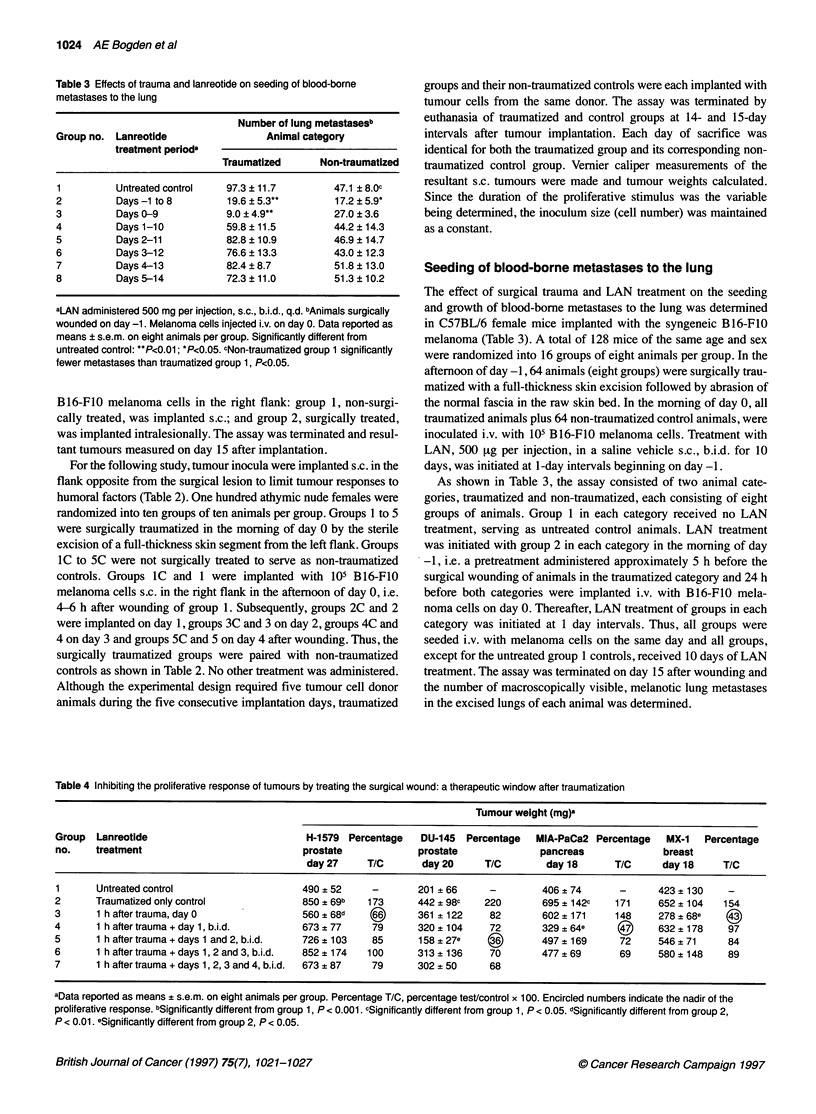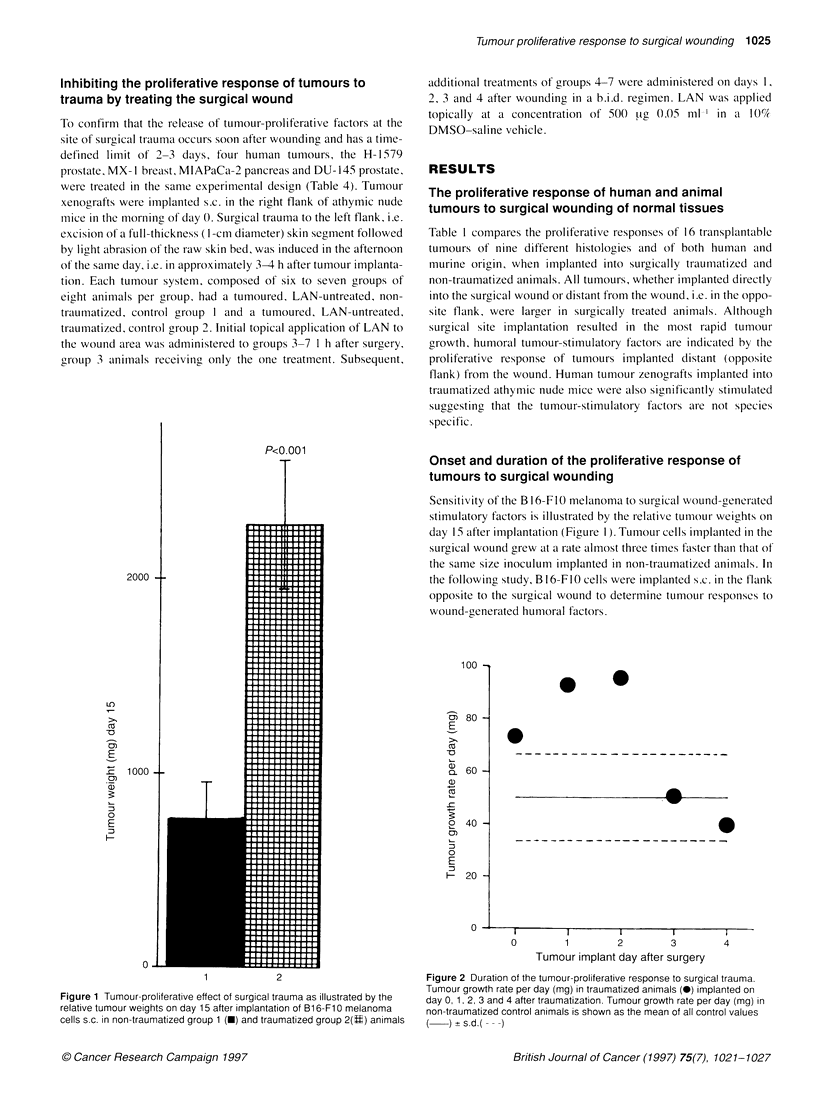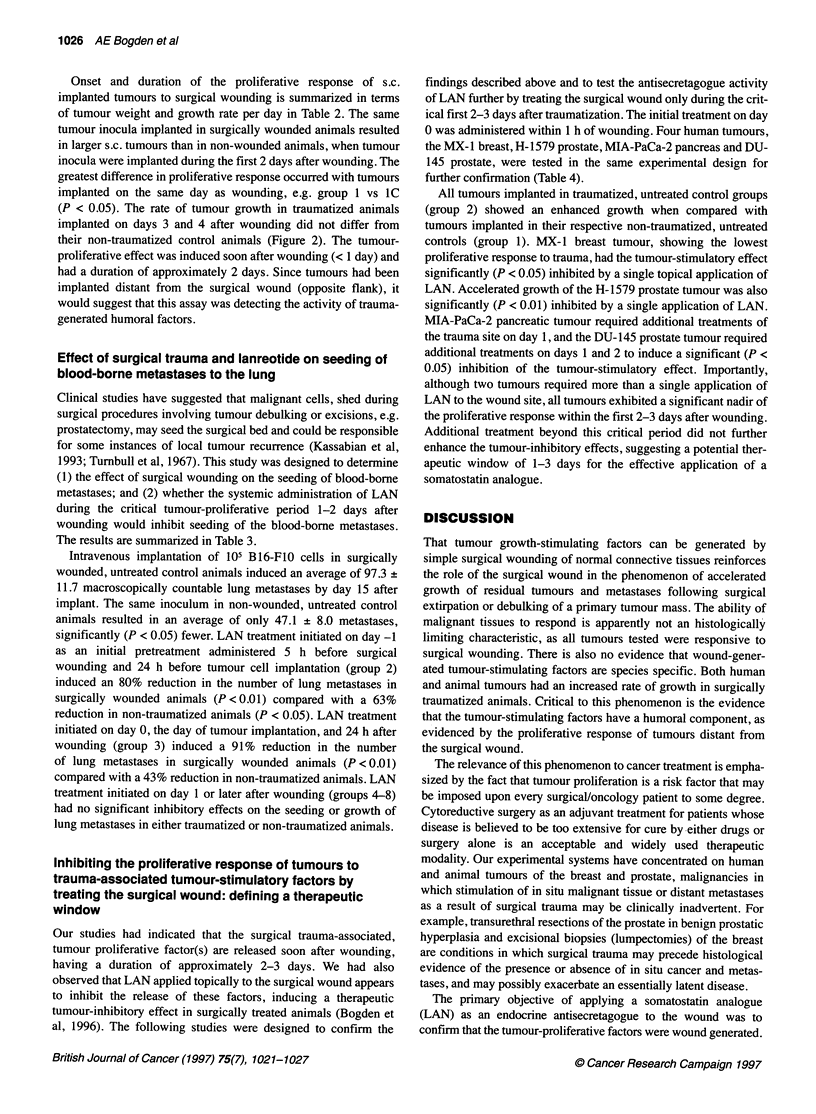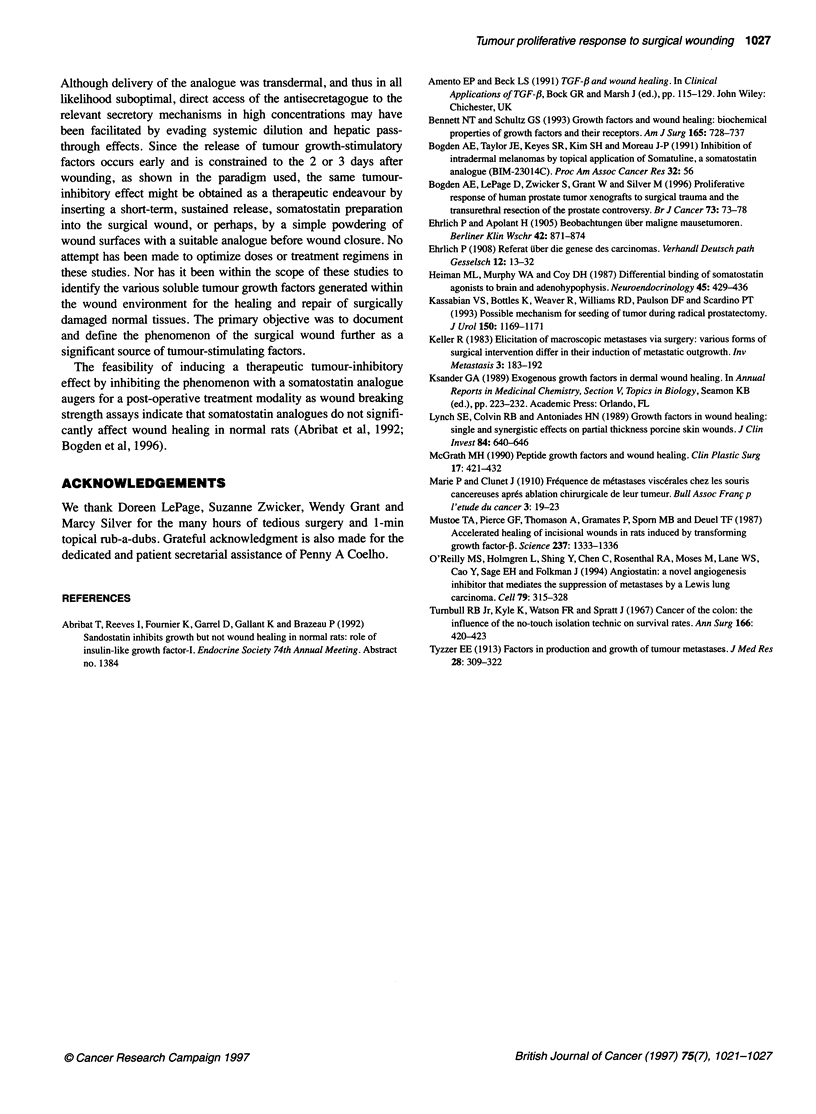# Proliferative response of human and animal tumours to surgical wounding of normal tissues: onset, duration and inhibition.

**DOI:** 10.1038/bjc.1997.175

**Published:** 1997

**Authors:** A. E. Bogden, J. P. Moreau, P. A. Eden

**Affiliations:** Biomeasure Inc., Milford, MA 01757-3650, USA.

## Abstract

Acceleration of secondary tumour growth and metastases following excision of a primary tumour has been attributed to the consequent removal of primary tumour-generated inhibitory factors. However, our studies have shown that surgical wounding of normal tissues significantly stimulated the growth of malignant tissues without the concomitant presence or excision of a tumour mass. A humoral stimulating component was indicated by the proliferative response of tumours and metastases distant from the surgical wound. All 16 human and murine tumours, of nine different histologies, showed a measurable acceleration of growth when implanted in surgically treated animals, suggesting that the ability of malignant tissue to respond to surgical wounding of normal tissue was not histologically or species specific. The proliferative surge of malignant tissues was detectable soon after wounding and had a duration of 2-3 days. The surgical wound as the source of the tumour-stimulating factor(s) was affirmed by the significant inhibition of tumour proliferative responses when a somatostatin analogue was applied topically to the surgical wound within 1 h of wounding, and/or during the critical tumour-stimulatory period of 1-2 days after wounding. A potential therapeutic window for reducing a risk factor that may be inadvertently imposed upon every surgical/oncology patient is indicated.


British Journal of Cancer (1997) 75(7), 1021–1027
© 1997 Cancer Research Campaign

### Proliferative response of human and animal tumours to surgical wounding of normal tissues: onset, duration and inhibition

**AE Bogden, J-P Moreau and PA Eden**

Biomeasure Inc., Milford, MA 01757–3650, USA

**Summary** Acceleration of secondary tumour growth and metastases following excision of a primary tumour has been attributed to the consequent removal of primary tumour-generated inhibitory factors. However, our studies have shown that surgical wounding of normal tissues significantly stimulated the growth of malignant tissues without the concomitant presence or excision of a tumour mass. A humoral stimulating component was indicated by the proliferative response of tumours and metastases distant from the surgical wound. All 16 human and murine tumours, of nine different histologies, showed a measurable acceleration of growth when implanted in surgically treated animals, suggesting that the ability of malignant tissue to respond to surgical wounding of normal tissue was not histologically or species specific. The proliferative surge of malignant tissues was detectable soon after wounding and had a duration of 2–3 days. The surgical wound as the source of the tumour-stimulating factor(s) was affirmed by the significant inhibition of tumour proliferative responses when a somatostatin analogue was applied topically to the surgical wound within 1 h of wounding, and/or during the critical tumour-stimulatory period of 1–2 days after wounding. A potential therapeutic window for reducing a risk factor that may be inadvertently imposed upon every surgical/oncology patient is indicated.

**Keywords:** surgical wounding; wound-generated tumour growth factor; tumour proliferative response; lanreotide; normal tissue trauma

Inhibition of tumour growth by tumour mass is a phenomenon recognized and repeatedly studied since the early 1900s (Ehrlich, 1908; Marie and Clunet, 1910; Tyzzer, 1913). Numerous reports, summarized more recently by Keller (1983) and O'Reilly et al (1994), have indicated that the presence of a primary tumour inhibits the growth rate of metastases or of a second tumour implant, and that removal or eradication of the primary tumour accelerates growth at secondary sites. Early explanations for apparent exacerbations of disease reflected Ehrlich's hypothesis of 'Athrepsia'; that any actively growing tumour removed certain specific nutritive material necessary for growth from the host animal (Ehrlich and Apolant, 1905). Subsequent explanations stressed surgical relief from growth-limiting factors, such as anatomical boundaries, anoxia and nutritional deficiencies, or that the immunological relation between host and tumour was somehow altered by surgery, thereby facilitating tumour escape. However, the underlying cause of the occasional explosive metastatic manifestation after resection of 'primary' malignancy remained in question. An alternative explanation, proposed by Keller (1983), suggested that soluble factors released by a tumour suppress the growth of tumours in other sites. Once the primary tumour is removed, its inhibitory influence is likewise abolished, permitting the unchecked growth of metastatic cells. More recently, O'Reilly et al (1994) demonstrated that inhibition of metastases by a primary tumour was mediated, in part, by a circulating angiogenesis inhibitor (angiostatin). They postulated that a primary tumour, while capable of stimulating angiogenesis in its own vascular bed by generating angiogenic stimulators in excess of angiogenesis inhibitors, the angiogenesis inhibitor, by virtue of its longer half-life in the circulation, reaches the vascular bed of a secondary tumour in excess of angiogenic stimulator escaping from the primary tumour or generated by the secondary tumour. While there is little question that, under the conditions hypothesized, excision of the primary tumour would abrogate the tumour-inhibitory effect, the role of the surgical wound, per se, in the phenomenon of accelerated growth of residual tumours and metastases following surgical extirpation or debulking of a primary tumour mass has been obscured by the interpretation that the proliferative response was the consequence of eliminating primary tumour-generated inhibitory factors.

There is now considerable evidence indicating that the overall process of healing and repair of surgically damaged tissue, including the necessary intercellular communication, is highly regulated in humans and other animals by a number of specific, soluble growth factors which are released within the wound environment and which appear to induce neovascularization, leucocyte chemotaxis, fibroblast proliferation, migration and deposition of collagen and other extracellular matrix molecules within the wounds. The growth factors that have been identified and isolated are, typically, specialized soluble proteins or polypeptides (McGrath, 1990; Ksander, 1989; Amento and Beck, 1991; Mustoe et al, 1987; Lynch et al, 1989; Bennett and Schultz, 1993). Thus, evidence of growth factors released at the site of trauma is extensive. With a more complex description of the various growth factors, it is increasingly apparent that the same mediators of cell growth and stromal synthesis are involved in malignancy, fetal growth and wound healing.

*Received 2 May 1996*
*Revised 8 October 1996*
*Accepted 16 October 1996*

*Correspondence to:* AE Bogden, Biomeasure Inc., 27 Maple Street, Milford, MA 01757–3650, USA

**Table 1** Proliferative response of human and animal tumours to surgical wounding of normal tissues

| Tumour designation | Species/strain of origin | Site of tumour implant[a] | Severity of trauma[b] | Percentage increased tumour weight[c] |
|---|---|---|---|---|
| Breast tumours | | | | |
| MX-1 | Human | Opposite flank | ++ | 155* |
| R-3230AC | Rat, Fischer | Opposite flank | ++ | 166* |
| Prostate tumours | | | | |
| H-1579 | Human | Surgical site | +++ | 930** |
| PC-3 | Human | Surgical site | +++ | 404** |
| DU-145 | Human | Surgical site | +++ | 383** |
| 11095 | Rat, Fischer | Opposite flank | ++ | 132* |
| 2PR121(D)1R | Rat, Noble | Opposite flank | ++ | 172 |
| 2PR121(D)1 | Rat, Noble | Opposite flank | ++ | 135 |
| Pancreatic tumours | | | | |
| MIA-PaCa | Human | Opposite flank | ++ | 305** |
| CAPAN-2 pancreatic | Human | Surgical site | ++ | 187* |
| Miscellaneous tumours | | | | |
| B16-melanoma | Mouse, C 57BL/6 | Surgical site | ++ | 298** |
| WR-6 lymphoma | Rat, Wistar–Furth | Opposite flank | + | 168 |
| FCB bladder | Mouse, C57BL/6 | Opposite flank | + | 160 |
| MPC-11 myeloma | Mouse, BALB/c | Opposite flank | + | 155 |
| CX-1 colon | Human | Opposite flank | + | 124 |
| SWARM sarcoma | Rat, Wistar–Furth | Opposite flank | + | 148 |

[a]Human tumours were tested as s.c. xenografts implanted in athymic nude mice. [b]Severity of trauma: +++, skin excision with abrasion; ++, skin excision; +, Alzet pump implantation. [c]Statistically significant according to Student's *t*-test: $^{*}P \leq 0.05$; $^{**}P \leq 0.01$. Tumour weight in traumatized animal (test)/tumour weight in non-traumatized animals (control) × 100

In our own studies, surgical traumatization (wounding) of normal connective tissues, such as excision of a segment of skin or abrasion of subcutaneous fascia, induced a significant proliferative response of human prostate tumour xenografts implanted in athymic nude mice (Bogden et al, 1996). Application of a somatostatin analogue, a neuroendocrine antisecretagogue, to the wound had an inhibitory effect on the proliferative response, suggesting the surgical wound as a source of tumour-stimulatory factors. Our primary objective has been to document further the role of the surgical wound, and thus to differentiate two phenomena: one that focuses on the surgical wounding of normal tissues and the resultant release of tumour-stimulating factors from the wound and the other as defined by O'Reilley et al (1994), which focuses on the surgical extirpation of a tumour mass and the coincident elimination of tumour-generated inhibitory factors. The feasibility of inducing a therapeutic antiproliferative effect by treating the surgical wound with a somatostatin analogue is demonstrated.

### MATERIAL AND METHODS

### Animals

Immunodeficient athymic nude mice (NCr-*nu*), used as recipients of human tumour xenografts for serial transplantation and testing, were received from Harlan, Madison, WI, USA, and housed in a pathogen-free biocontainment facility Tumour donor and athymic test animals were maintained ad libitum on an irradiated, Harlan Teklad LM-485 mouse/rat diet. The immunocompetent three rat and two mouse strains listed in Table 1, used as recipients of syngeneic tumour grafts, were maintained ad libitum on the standard Formulab diet 5008. All procedures were performed in compliance with the Guide for the Care and use of Laboratory Animals, NIH Publication No. 86–23, revised 1985, and enhanced Standard Operating Procedures on humane use and care of laboratory animals in a pathogen-free barrier facility maintained at our laboratories.

### Tumour sources

The PC-3 and DU-145 human prostate adenocarcinomas were obtained from the American Type Culture Collection, Rockville, MD, USA, as in vitro maintained cell culture systems. They were adapted to in vivo transplantation in our laboratory. The H-1579 human prostate tumour was established in vivo transplantation directly as a primary explant. This tumour, as well as all other human and animal tumours used in this study, was obtained from and maintained by the Breast Cancer Animal and Human Tumor Bank, Biomeasure Inc., Milford, MA, USA. The Tumor Bank has subsequently been transferred to the DCT Tumor Repository, maintained as a service facility by the Biological Testing Branch, Division of Cancer Treatment, National Cancer Institute, Frederick Cancer Research and Development Center, Frederick, MD, USA.

### Tumour implantations

Both human and animal tumour systems were carried in serial transplantation by subcutaneous (s.c.) implantation of a 2–3 $mm^3$ mince into the right flank. Tumours were transplanted from donor animals at the mid-log phase of growth. Human tumours served as xenografts in athymic nude mice and murine tumours were implanted in their syngeneic strain of origin. Tumour grafts were implanted directly into the trauma site under the suture lines (intralesional) or in the flank contralateral to the surgical wound when topical treatment was to be applied to the trauma. The 16 tumour lines included in this study are listed in Table 1. The murine melanoma B16-F10 was selected as the initial experimental test system because of its growth and morphological characteristics resembling cutaneous melanoma. When implanted s.c. or i.d. into athymic nude mice, black, cutaneous lesions are clearly discernible within 3 days (Bogden et al, 1991). Cells injected i.v. result in black, macroscopically visible metastases. An inoculum

© Cancer Research Campaign 1997

of $10^5$ cells was used for implanting both C57BL/6 and athymic nude female mice.

Tumours were measured two or three times weekly with Vernier calipers and tumour weight (mg) was calculated from tumour dimensions (mm x mm) following the formula of a prolate ellipsoid:

$L \times W^2/2$

Where $L$ is the longer of the two measurements and the first value recorded. Levels of statistical significance were determined with Student's $t$-test.

### Surgical trauma

Severity of surgical trauma varied in degree and is classified as follows: + + +, skin excision with abrasion of subcutaneous fascia at the surgical site; + +, skin excision only; and +, Alzet pump implantation s.c.

### *Skin excision*

A section of skin, full thickness, approximately 12 mm in diameter was excised from the right or left flank under sterile conditions and under light ether anaesthesia. Wound edges were immediately approximated and closed with Michel clamps.

### *Skin excision with abrasion*

A 12-mm-diameter, full-thickness skin graft was excised from the right or left flank under sterile conditions. The raw graft bed was then traumatized by abrading with a burred needle. Abrasion was accomplished by carefully, but firmly, drawing the burred needle the full length of the exposed subcutaneous tissues four times and then again at right angles four times. Bleeding was minimal and the surgical wound was immediately closed with Michel clamps. Animals were anaesthetized with intraperitoneally (i.p.) administered 4% chloral hydrate.

### Lanreotide (LAN) treatment

Lanreotide (BIM-23014C, Somatuline) having the structure [D-β-Nal-Cys-Tyr-D-Trp-Lys-Val-Cys- Thr-$NH_2$] acetate is a long-acting octapeptide analogue of somatostatin (SRIF), a neuroendocrine antisecretagogue (Heiman et al, 1987). To enhance transdermal delivery, it was administered at a concentration of 500 μg 0.05 $ml^{-1}$ in either a 10%, 25% or 50% dimethylsulphoxide (DMSO) – saline vehicle. Treatment consisted of a 0.05-ml drop applied topically to the surgically treated area. Lanreotide (LAN) was then gently rubbed onto the surgical area and around the wound clips for 1 min with a latex-gloved finger. The DMSO – saline vehicle was administered similarly.

### Timing of assay endpoint

A comparison of tumour growth between test and control animals was made after allowing for the time required for a tumour proliferative response to be translated into a relative increase in tumour mass. This size difference between tumours, resulting from the same size inocula implanted in intact and surgically wounded animals, becomes evident during log phase of measurable tumour growth. Since tumour lines differ in growth rate, timing of assay end point differed between assays. A prerequisite is to implant the tumour inocula during the critical 1–3 days after wounding. As the proliferative response occurs as a proliferative surge soon after wounding, the resultant difference in tumour mass, measurable during log phase of growth, resembles that of an initial difference in inoculum size.

### Proliferative response of human and animal tumours to surgical trauma

This study was, in essence, a screening of the proliferative responses of human and animal tumours to acquire additional experimental evidence concerning the histological and species specificity of the wound-generated tumour-stimulatory factors. Sixteen transplantable tumours of nine different histologies and of both human and murine origin were implanted into surgically traumatized and non-traumatized animals. Although the severity of the surgical wound varied in degree between assays, within each assay grafts of identical size were implanted in both surgically traumatized, as well as non-traumatized, hosts (Table 1). The percentage increase in tumour weight in traumatized (test) over that in non-traumatized (control) animals was determined during mid or log phase of growth as the percentage test/control x 100.

### Determining the onset and duration of the proliferative response of tumours to surgical trauma

The effect of surgical wounding of normal tissues on the growth of B16-F10 cells implanted s.c. in athymic nude females was determined by excision of a full-thickness skin segment from the right flank on day 0. On day 1, all animals were implanted with $10^5$

**Table 2** Onset and duration of the proliferative response of tumours to surgical trauma

| Group no. | Treatment | Days after implant | Tumour weight[a] (mg) | Growth rate per day (mg) |
|---|---|---|---|---|
| 1C | No surgery control. Tumour implanted s.c., right flank, day 0 | 14 | 454 ± 76 | 32.5 |
| 1 | Surgery left flank, day 0. Tumour implanted s.c., right flank, day 0 | 14 | 1031 ± 112[b] | 73.6 |
| 2C | No surgery control. Tumour implanted s.c., right flank, day 1 | 15 | 972 ± 232 | 64.8 |
| 2 | Surgery left flank, day 0. Tumour implanted s.c., right flank, day 1 | 15 | 1391 ± 166 | 92.7 |
| 3C | No surgery control. Tumour implanted s.c., right flank, day 2 | 14 | 813 ± 268 | 58.1 |
| 3 | Surgery left flank, day 0. Tumour implanted s.c., right flank, day 2 | 14 | 1333 ± 244 | 95.2 |
| 4C | No surgery control. Tumour implanted s.c., right flank day 3 | 15 | 950 ± 185 | 63.3 |
| 4 | Surgery left flank, day 0. Tumour implanted s.c., right flank, day 3 | 15 | 764 ± 166 | 50.9 |
| 5C | No surgery control. Tumour implanted s.c., right flank, day 4 | 14 | 448 ± 111 | 31.9 |
| 5 | Surgery left flank, day 0. Tumour implanted s.c., right flank, day 4 | 14 | 558 ± 200 | 39.8 |

[a]Data reported as means ± s.e.m. on ten animals per group. [b]Significantly larger than non-traumatized control, $P \leq 0.05$.

© Cancer Research Campaign 1997

**Table 3** Effects of trauma and lanreotide on seeding of blood-borne metastases to the lung

| Group no. | Lanreotide treatment period[a] | Number of lung metastases[b] Animal category | |
|---|---|---|---|
| | | Traumatized | Non-traumatized |
| 1 | Untreated control | 97.3 ± 11.7 | 47.1 ± 8.0[c] |
| 2 | Days –1 to 8 | 19.6 ± 5.3** | 17.2 ± 5.9* |
| 3 | Days 0–9 | 9.0 ± 4.9** | 27.0 ± 3.6 |
| 4 | Days 1–10 | 59.8 ± 11.5 | 44.2 ± 14.3 |
| 5 | Days 2–11 | 82.8 ± 10.9 | 46.9 ± 14.7 |
| 6 | Days 3–12 | 76.6 ± 13.3 | 43.0 ± 12.3 |
| 7 | Days 4–13 | 82.4 ± 8.7 | 51.8 ± 13.0 |
| 8 | Days 5–14 | 72.3 ± 11.0 | 51.3 ± 10.2 |

[a]LAN administered 500 mg per injection, s.c., b.i.d., q.d. [b]Animals surgically wounded on day –1. Melanoma cells injected i.v. on day 0. Data reported as means ± s.e.m. on eight animals per group. Significantly different from untreated control: ***P*<0.01; **P*<0.05. [c]Non-traumatized group 1 significantly fewer metastases than traumatized group 1, *P*<0.05.

B16-F10 melanoma cells in the right flank: group 1, non-surgically treated, was implanted s.c.; and group 2, surgically treated, was implanted intralesionally. The assay was terminated and resultant tumours measured on day 15 after implantation.

For the following study, tumour inocula were implanted s.c. in the flank opposite from the surgical lesion to limit tumour responses to humoral factors (Table 2). One hundred athymic nude females were randomized into ten groups of ten animals per group. Groups 1 to 5 were surgically traumatized in the morning of day 0 by the sterile excision of a full-thickness skin segment from the left flank. Groups 1C to 5C were not surgically treated to serve as non-traumatized controls. Groups 1C and 1 were implanted with $10^5$ B16-F10 melanoma cells s.c. in the right flank in the afternoon of day 0, i.e. 4–6 h after wounding of group 1. Subsequently, groups 2C and 2 were implanted on day 1, groups 3C and 3 on day 2, groups 4C and 4 on day 3 and groups 5C and 5 on day 4 after wounding. Thus, the surgically traumatized groups were paired with non-traumatized controls as shown in Table 2. No other treatment was administered. Although the experimental design required five tumour cell donor animals during the five consecutive implantation days, traumatized groups and their non-traumatized controls were each implanted with tumour cells from the same donor. The assay was terminated by euthanasia of traumatized and control groups at 14- and 15-day intervals after tumour implantation. Each day of sacrifice was identical for both the traumatized group and its corresponding non-traumatized control group. Vernier caliper measurements of the resultant s.c. tumours were made and tumour weights calculated. Since the duration of the proliferative stimulus was the variable being determined, the inoculum size (cell number) was maintained as a constant.

### Seeding of blood-borne metastases to the lung

The effect of surgical trauma and LAN treatment on the seeding and growth of blood-borne metastases to the lung was determined in C57BL/6 female mice implanted with the syngeneic B16-F10 melanoma (Table 3). A total of 128 mice of the same age and sex were randomized into 16 groups of eight animals per group. In the afternoon of day –1, 64 animals (eight groups) were surgically traumatized with a full-thickness skin excision followed by abrasion of the normal fascia in the raw skin bed. In the morning of day 0, all traumatized animals plus 64 non-traumatized control animals, were inoculated i.v. with $10^5$ B16-F10 melanoma cells. Treatment with LAN, 500 μg per injection, in a saline vehicle s.c., b.i.d. for 10 days, was initiated at 1-day intervals beginning on day –1.

As shown in Table 3, the assay consisted of two animal categories, traumatized and non-traumatized, each consisting of eight groups of animals. Group 1 in each category received no LAN treatment, serving as untreated control animals. LAN treatment was initiated with group 2 in each category in the morning of day –1, i.e. a pretreatment administered approximately 5 h before the surgical wounding of animals in the traumatized category and 24 h before both categories were implanted i.v. with B16-F10 melanoma cells on day 0. Thereafter, LAN treatment of groups in each category was initiated at 1 day intervals. Thus, all groups were seeded i.v. with melanoma cells on the same day and all groups, except for the untreated group 1 controls, received 10 days of LAN treatment. The assay was terminated on day 15 after wounding and the number of macroscopically visible, melanotic lung metastases in the excised lungs of each animal was determined.

**Table 4** Inhibiting the proliferative response of tumours by treating the surgical wound: a therapeutic window after traumatization

| Group no. | Lanreotide treatment | Tumour weight (mg)[a] H-1579 prostate day 27 | Percentage T/C | DU-145 prostate day 20 | Percentage T/C | MIA-PaCa2 pancreas day 18 | Percentage T/C | MX-1 breast day 18 | Percentage T/C |
|---|---|---|---|---|---|---|---|---|---|
| 1 | Untreated control | 490 ± 52 | – | 201 ± 66 | – | 406 ± 74 | – | 423 ± 130 | – |
| 2 | Traumatized only control | 850 ± 69[b] | 173 | 442 ± 98[c] | 220 | 695 ± 142[c] | 171 | 652 ± 104 | 154 |
| 3 | 1 h after trauma, day 0 | 560 ± 68[d] | (66) | 361 ± 122 | 82 | 602 ± 171 | 148 | 278 ± 68[e] | (43) |
| 4 | 1 h after trauma + day 1, b.i.d. | 673 ± 77 | 79 | 320 ± 104 | 72 | 329 ± 64[e] | (47) | 632 ± 178 | 97 |
| 5 | 1 h after trauma + days 1 and 2, b.i.d. | 726 ± 103 | 85 | 158 ± 27[e] | (36) | 497 ± 169 | 72 | 546 ± 71 | 84 |
| 6 | 1 h after trauma + days 1, 2 and 3, b.i.d. | 852 ± 174 | 100 | 313 ± 136 | 70 | 477 ± 69 | 69 | 580 ± 148 | 89 |
| 7 | 1 h after trauma + days 1, 2, 3 and 4, b.i.d. | 673 ± 87 | 79 | 302 ± 50 | 68 | | | | |

[a]Data reported as means ± s.e.m. on eight animals per group. Percentage T/C, percentage test/control × 100. Encircled numbers indicate the nadir of the proliferative response. [b]Significantly different from group 1, *P* < 0.001. [c]Significantly different from group 1, *P* < 0.05. [d]Significantly different from group 2, *P* < 0.01. [e]Significantly different from group 2, *P* < 0.05.

*© Cancer Research Campaign 1997*

### Inhibiting the proliferative response of tumours to trauma by treating the surgical wound

To confirm that the release of tumour-proliferative factors at the site of surgical trauma occurs soon after wounding and has a time-defined limit of 2–3 days, four human tumours, the H-1579 prostate, MX-1 breast, MIAPaCa-2 pancreas and DU-145 prostate, were treated in the same experimental design (Table 4). Tumour xenografts were implanted s.c. in the right flank of athymic nude mice in the morning of day 0. Surgical trauma to the left flank, i.e. excision of a full-thickness (1-cm diameter) skin segment followed by light abrasion of the raw skin bed, was induced in the afternoon of the same day, i.e. in approximately 3–4 h after tumour implantation. Each tumour system, composed of six to seven groups of eight animals per group, had a tumoured, LAN-untreated, non-traumatized, control group 1 and a tumoured, LAN-untreated, traumatized, control group 2. Initial topical application of LAN to the wound area was administered to groups 3–7 1 h after surgery, group 3 animals receiving only the one treatment. Subsequent, additional treatments of groups 4–7 were administered on days 1, 2, 3 and 4 after wounding in a b.i.d. regimen. LAN was applied topically at a concentration of 500 μg 0.05 $ml^{-1}$ in a 10% DMSO–saline vehicle.

### RESULTS

### The proliferative response of human and animal tumours to surgical wounding of normal tissues

Table 1 compares the proliferative responses of 16 transplantable tumours of nine different histologies and of both human and murine origin, when implanted into surgically traumatized and non-traumatized animals. All tumours, whether implanted directly into the surgical wound or distant from the wound, i.e. in the opposite flank, were larger in surgically treated animals. Although surgical site implantation resulted in the most rapid tumour growth, humoral tumour-stimulatory factors are indicated by the proliferative response of tumours implanted distant (opposite flank) from the wound. Human tumour zenografts implanted into traumatized athymic nude mice were also significantly stimulated suggesting that the tumour-stimulatory factors are not species specific.

### Onset and duration of the proliferative response of tumours to surgical wounding

Sensitivity of the B16-F10 melanoma to surgical wound-generated stimulatory factors is illustrated by the relative tumour weights on day 15 after implantation (Figure 1). Tumour cells implanted in the surgical wound grew at a rate almost three times faster than that of the same size inoculum implanted in non-traumatized animals. In the following study, B16-F10 cells were implanted s.c. in the flank opposite to the surgical wound to determine tumour responses to wound-generated humoral factors.

**Figure 1** Tumour-proliferative effect of surgical trauma as illustrated by the relative tumour weights on day 15 after implantation of B16-F10 melanoma cells s.c. in non-traumatized group 1 (■) and traumatized group 2(⊞) animals

**Figure 2** Duration of the tumour-proliferative response to surgical trauma. Tumour growth rate per day (mg) in traumatized animals (●) implanted on day 0, 1, 2, 3 and 4 after traumatization. Tumour growth rate per day (mg) in non-traumatized control animals is shown as the mean of all control values (——) ± s.d.( - - -)

Onset and duration of the proliferative response of s.c. implanted tumours to surgical wounding is summarized in terms of tumour weight and growth rate per day in Table 2. The same tumour inocula implanted in surgically wounded animals resulted in larger s.c. tumours than in non-wounded animals, when tumour inocula were implanted during the first 2 days after wounding. The greatest difference in proliferative response occurred with tumours implanted on the same day as wounding, e.g. group 1 vs 1C ($P < 0.05$). The rate of tumour growth in traumatized animals implanted on days 3 and 4 after wounding did not differ from their non-traumatized control animals (Figure 2). The tumour-proliferative effect was induced soon after wounding (< 1 day) and had a duration of approximately 2 days. Since tumours had been implanted distant from the surgical wound (opposite flank), it would suggest that this assay was detecting the activity of trauma-generated humoral factors.

### Effect of surgical trauma and lanreotide on seeding of blood-borne metastases to the lung

Clinical studies have suggested that malignant cells, shed during surgical procedures involving tumour debulking or excisions, e.g. prostatectomy, may seed the surgical bed and could be responsible for some instances of local tumour recurrence (Kassabian et al, 1993; Turnbull et al, 1967). This study was designed to determine (1) the effect of surgical wounding on the seeding of blood-borne metastases; and (2) whether the systemic administration of LAN during the critical tumour-proliferative period 1–2 days after wounding would inhibit seeding of the blood-borne metastases. The results are summarized in Table 3.

Intravenous implantation of $10^5$ B16-F10 cells in surgically wounded, untreated control animals induced an average of 97.3 ± 11.7 macroscopically countable lung metastases by day 15 after implant. The same inoculum in non-wounded, untreated control animals resulted in an average of only 47.1 ± 8.0 metastases, significantly ($P < 0.05$) fewer. LAN treatment initiated on day –1 as an initial pretreatment administered 5 h before surgical wounding and 24 h before tumour cell implantation (group 2) induced an 80% reduction in the number of lung metastases in surgically wounded animals ($P < 0.01$) compared with a 63% reduction in non-traumatized animals ($P < 0.05$). LAN treatment initiated on day 0, the day of tumour implantation, and 24 h after wounding (group 3) induced a 91% reduction in the number of lung metastases in surgically wounded animals ($P < 0.01$) compared with a 43% reduction in non-traumatized animals. LAN treatment initiated on day 1 or later after wounding (groups 4–8) had no significant inhibitory effects on the seeding or growth of lung metastases in either traumatized or non-traumatized animals.

### Inhibiting the proliferative response of tumours to trauma-associated tumour-stimulatory factors by treating the surgical wound: defining a therapeutic window

Our studies had indicated that the surgical trauma-associated, tumour proliferative factor(s) are released soon after wounding, having a duration of approximately 2–3 days. We had also observed that LAN applied topically to the surgical wound appears to inhibit the release of these factors, inducing a therapeutic tumour-inhibitory effect in surgically treated animals (Bogden et al, 1996). The following studies were designed to confirm the findings described above and to test the antisecretagogue activity of LAN further by treating the surgical wound only during the critical first 2–3 days after traumatization. The initial treatment on day 0 was administered within 1 h of wounding. Four human tumours, the MX-1 breast, H-1579 prostate, MIA-PaCa-2 pancreas and DU-145 prostate, were tested in the same experimental design for further confirmation (Table 4).

All tumours implanted in traumatized, untreated control groups (group 2) showed an enhanced growth when compared with tumours implanted in their respective non-traumatized, untreated controls (group 1). MX-1 breast tumour, showing the lowest proliferative response to trauma, had the tumour-stimulatory effect significantly ($P < 0.05$) inhibited by a single topical application of LAN. Accelerated growth of the H-1579 prostate tumour was also significantly ($P < 0.01$) inhibited by a single application of LAN. MIA-PaCa-2 pancreatic tumour required additional treatments of the trauma site on day 1, and the DU-145 prostate tumour required additional treatments on days 1 and 2 to induce a significant ($P < 0.05$) inhibition of the tumour-stimulatory effect. Importantly, although two tumours required more than a single application of LAN to the wound site, all tumours exhibited a significant nadir of the proliferative response within the first 2–3 days after wounding. Additional treatment beyond this critical period did not further enhance the tumour-inhibitory effects, suggesting a potential therapeutic window of 1–3 days for the effective application of a somatostatin analogue.

### DISCUSSION

That tumour growth-stimulating factors can be generated by simple surgical wounding of normal connective tissues reinforces the role of the surgical wound in the phenomenon of accelerated growth of residual tumours and metastases following surgical extirpation or debulking of a primary tumour mass. The ability of malignant tissues to respond is apparently not an histologically limiting characteristic, as all tumours tested were responsive to surgical wounding. There is also no evidence that wound-generated tumour-stimulating factors are species specific. Both human and animal tumours had an increased rate of growth in surgically traumatized animals. Critical to this phenomenon is the evidence that the tumour-stimulating factors have a humoral component, as evidenced by the proliferative response of tumours distant from the surgical wound.

The relevance of this phenomenon to cancer treatment is emphasized by the fact that tumour proliferation is a risk factor that may be imposed upon every surgical/oncology patient to some degree. Cytoreductive surgery as an adjuvant treatment for patients whose disease is believed to be too extensive for cure by either drugs or surgery alone is an acceptable and widely used therapeutic modality. Our experimental systems have concentrated on human and animal tumours of the breast and prostate, malignancies in which stimulation of in situ malignant tissue or distant metastases as a result of surgical trauma may be clinically inadvertent. For example, transurethral resections of the prostate in benign prostatic hyperplasia and excisional biopsies (lumpectomies) of the breast are conditions in which surgical trauma may precede histological evidence of the presence or absence of in situ cancer and metastases, and may possibly exacerbate an essentially latent disease.

The primary objective of applying a somatostatin analogue (LAN) as an endocrine antisecretagogue to the wound was to confirm that the tumour-proliferative factors were wound generated.

© Cancer Research Campaign 1997

Although delivery of the analogue was transdermal, and thus in all likelihood suboptimal, direct access of the antisecretagogue to the relevant secretory mechanisms in high concentrations may have been facilitated by evading systemic dilution and hepatic pass-through effects. Since the release of tumour growth-stimulatory factors occurs early and is constrained to the 2 or 3 days after wounding, as shown in the paradigm used, the same tumour-inhibitory effect might be obtained as a therapeutic endeavour by inserting a short-term, sustained release, somatostatin preparation into the surgical wound, or perhaps, by a simple powdering of wound surfaces with a suitable analogue before wound closure. No attempt has been made to optimize doses or treatment regimens in these studies. Nor has it been within the scope of these studies to identify the various soluble tumour growth factors generated within the wound environment for the healing and repair of surgically damaged normal tissues. The primary objective was to document and define the phenomenon of the surgical wound further as a significant source of tumour-stimulating factors.

The feasibility of inducing a therapeutic tumour-inhibitory effect by inhibiting the phenomenon with a somatostatin analogue augers for a post-operative treatment modality as wound breaking strength assays indicate that somatostatin analogues do not significantly affect wound healing in normal rats (Abribat et al, 1992; Bogden et al, 1996).

### ACKNOWLEDGEMENTS

We thank Doreen LePage, Suzanne Zwicker, Wendy Grant and Marcy Silver for the many hours of tedious surgery and 1-min topical rub-a-dubs. Grateful acknowledgment is also made for the dedicated and patient secretarial assistance of Penny A Coelho.

### REFERENCES

Abribat T, Reeves I, Fournier K, Garrel D, Gallant K and Brazeau P (1992) Sandostatin inhibits growth but not wound healing in normal rats: role of insulin-like growth factor-I. *Endocrine Society 74th Annual Meeting*. Abstract no. 1384

Amento EP and Beck LS (1991) *TGF-β and wound healing*. In *Clinical Applications of TGF-β*, Bock GR and Marsh J (ed.), pp. 115–129. John Wiley: Chichester, UK

Bennett NT and Schultz GS (1993) Growth factors and wound healing: biochemical properties of growth factors and their receptors. *Am J Surg* **165**: 728–737

Bogden AE, Taylor JE, Keyes SR, Kim SH and Moreau J-P (1991) Inhibition of intradermal melanomas by topical application of Somatuline, a somatostatin analogue (BIM-23014C). *Proc Am Assoc Cancer Res* **32**: 56

Bogden AE, LePage D, Zwicker S, Grant W and Silver M (1996) Proliferative response of human prostate tumor xenografts to surgical trauma and the transurethral resection of the prostate controversy. *Br J Cancer* **73**: 73–78

Ehrlich P and Apolant H (1905) Beobachtungen über maligne mausetumoren. *Berliner Klin Wschr* **42**: 871–874

Ehrlich P (1908) Referat über die genese des carcinomas. *Verhandl Deutsch path Gesselsch* **12**: 13–32

Heiman ML, Murphy WA and Coy DH (1987) Differential binding of somatostatin agonists to brain and adenohypophysis. *Neuroendocrinology* **45**: 429–436

Kassabian VS, Bottles K, Weaver R, Williams RD, Paulson DF and Scardino PT (1993) Possible mechanism for seeding of tumor during radical prostatectomy. *J Urol* **150**: 1169–1171

Keller R (1983) Elicitation of macroscopic metastases via surgery: various forms of surgical intervention differ in their induction of metastatic outgrowth. *Inv Metastasis* **3**: 183–192

Ksander GA (1989) Exogenous growth factors in dermal wound healing. In *Annual Reports in Medicinal Chemistry, Section V, Topics in Biology*, Seamon KB (ed.), pp. 223–232. Academic Press: Orlando, FL

Lynch SE, Colvin RB and Antoniades HN (1989) Growth factors in wound healing: single and synergistic effects on partial thickness porcine skin wounds. *J Clin Invest* **84**: 640–646

McGrath MH (1990) Peptide growth factors and wound healing. *Clin Plastic Surg* **17**: 421–432

Marie P and Clunet J (1910) Fréquence de métastases viscérales chez les souris cancereuses aprés ablation chirurgicale de leur tumeur. *Bull Assoc Franç p l'etude du cancer* **3**: 19–23

Mustoe TA, Pierce GF, Thomason A, Gramates P, Sporn MB and Deuel TF (1987) Accelerated healing of incisional wounds in rats induced by transforming growth factor-β. *Science* **237**: 1333–1336

O'Reilly MS, Holmgren L, Shing Y, Chen C, Rosenthal RA, Moses M, Lane WS, Cao Y, Sage EH and Folkman J (1994) Angiostatin: a novel angiogenesis inhibitor that mediates the suppression of metastases by a Lewis lung carcinoma. *Cell* **79**: 315–328

Turnbull RB Jr, Kyle K, Watson FR and Spratt J (1967) Cancer of the colon: the influence of the no-touch isolation technic on survival rates. *Ann Surg* **166**: 420–423

Tyzzer EE (1913) Factors in production and growth of tumour metastases. *J Med Res* **28**: 309–322

© Cancer Research Campaign 1997